# Decreasing suicide mortality in Hungary – What are the main causes?

**DOI:** 10.1192/j.eurpsy.2022.342

**Published:** 2022-09-01

**Authors:** Z. Rihmer, P. Dome, X. Gonda, Z. Bélteczki, A. Németh, S. Szilágyi, J. Balazs

**Affiliations:** 1 Semmelweis University, Department Of Psychiatry And Psychotherapy, Budapest, Hungary; 2 Sántha Kálmán Psychiatric Hospital, I. Department, Nagykálló, Hungary; 3 National Institute of Mental Health, Neurology and Neurosurgery, Nyírő Gyula National Institute Of Psychiatry And Addictions, Budapest, Hungary; 4 Peterfy Sándor Hospital, Department Of Crisis Intervention And Psychiatry, Budapest, Hungary; 5 Eotvos Lorant University, Department Of Developmental And Clinical Child Psychology, Budapest, Hungary

**Keywords:** Hungary, suicide, mortality

## Abstract

**Introduction:**

Depression and suicidal behaviour are major public health problems everywhere but particularly in Hungary where until 2000 the suicide rate was among the highest in the world.

**Objectives:**

To analyse the possible causes of declining national suicide rate of Hungary.

**Methods:**

Review of the scientific literature on Hungarian suicide scene published in the last 40 years.

**Results:**

The peak of Hungarian national suicide rate was in 1985 (46/100.000) but due to a steady and continuous, year by year decline, in 2019 it was only 16/100.000, which represents a more than 65% decrease. Rate of unrecognised/untreated mood disorders, availability of health/psychiatric care, antidepressant and lithium prescription, unemployment, smoking and alcohol consumption as well as lithium and arsenic contents of drinking water were the most investigated possible determinants of suicide mortality of the country. More widespread and effective treatment of psychiatric/mood disorder patients, decreased rate of unemployment and smoking as well as the continuously improving living standards were the most important contributors to the great decline of the national suicide rate. However, in 2020 – the first year of the COVID-19 pandemic – the national suicide rate rose by 16%, which was almost totally accounted for by the increase of suicides among males.

**Conclusions:**

Suicidal behaviour is preventable in many cases, but as it is a complex, multicausal phenomenon, its prevention should involve several medical/psychiatric, psychosocial and community interventions.

**Disclosure:**

No significant relationships.

